# Neutralizing Anti-Cytokine Autoantibodies Against Interferon-α in Immunodysregulation Polyendocrinopathy Enteropathy X-Linked

**DOI:** 10.3389/fimmu.2018.00544

**Published:** 2018-03-29

**Authors:** Jacob M. Rosenberg, Maria E. Maccari, Federica Barzaghi, Eric J. Allenspach, Claudio Pignata, Giovanna Weber, Troy R. Torgerson, Paul J. Utz, Rosa Bacchetta

**Affiliations:** ^1^Department of Medicine, Massachusetts General Hospital, Harvard Medical School, Boston, MA, United States; ^2^Division of Immunology and Rheumatology, Department of Medicine, Stanford University School of Medicine, Stanford, CA, United States; ^3^Center for Chronic Immunodeficiency, Faculty of Medicine, University Medical Center, University of Freiburg, Freiburg, Germany; ^4^San Raffaele Telethon Institute for Gene Therapy, Division of Regenerative Medicine, Stem Cells and Gene Therapy, San Raffaele Scientific Institute, Milan, Italy; ^5^University of Washington School of Medicine and Seattle Children’s Hospital, Seattle, WA, United States; ^6^Pediatric Section, Department of Translational Medical Sciences, University of Naples Federico II, Naples, Italy; ^7^Department of Pediatrics, IRCCS San Raffaele Hospital, Vita-Salute San Raffaele University, Milan, Italy; ^8^Institute for Immunity, Transplantation, and Infection, Stanford University School of Medicine, Stanford, CA, United States; ^9^Department of Pediatric Stem Cell Transplantation and Regenerative Medicine, Stanford School of Medicine, Stanford, CA, United States

**Keywords:** anti-cytokine autoantibodies, interferon-alpha, immunodysregulation polyendocrinopathy enteropathy X-linked, autoimmune polyendocrine syndrome type I, protein microarrays

## Abstract

Anti-cytokine autoantibodies (ACAAs) have been described in a growing number of primary immunodeficiencies with autoimmune features, including autoimmune polyendocrine syndrome type I (APS-1), a prototypical disease of defective T cell-mediated central tolerance. Whether defects in peripheral tolerance lead to similar ACAAs is unknown. Immunodysregulation polyendocrinopathy enteropathy X-linked (IPEX) is caused by mutations in *FOXP3*, a master regulator of T regulatory cells (T_reg_), and consequently results in defective T cell-mediated peripheral tolerance. Unique autoantibodies have previously been described in IPEX. To test the hypothesis that ACAAs are present in IPEX, we designed and fabricated antigen microarrays. We discovered elevated levels of IgG ACAAs against interferon-α (IFN-α) in a cohort of IPEX patients. Serum from IPEX patients blocked IFN-α signaling *in vitro* and blocking activity was tightly correlated with ACAA titer. To show that blocking activity was mediated by IgG and not other serum factors, we purified IgG and showed that blocking activity was contained entirely in the immunoglobulin fraction. We also screened for ACAAs against IFN-α in a second geographically distinct cohort. In these samples, ACAAs against IFN-α were elevated in a *post hoc* analysis. In summary, we report the discovery of ACAAs against IFN-α in IPEX, an experiment of nature demonstrating the important role of peripheral T cell tolerance.

## Introduction

Anti-cytokine autoantibodies (ACAAs) have been described in a growing number of immunodeficiencies (1, 2). Additionally, ACAAs have been identified in the serum of patients with autoimmune disease and healthy donors (3–6). However, the prevalence of ACAAs in many human diseases, and their contributions to phenotype, remain understudied.

Human type I interferons (IFNs) are a cytokine family with antiviral activity whose members include 13 interferon-α (IFN-α) proteins, IFN-β, and several poorly defined gene products (7). High titer ACAAs against IFN-α and other type I IFNs are strikingly specific to the disease autoimmune polyendocrine syndrome type I (APS-1), although they have also been described in the following rare conditions with varying penetrance: patients with thymoma, patients with hypomorphic *RAG1* or *RAG2* mutations, and in a patient with an *NFKB2* mutation (8–10). Although there is debate as to whether the 13 different IFN-α paralogs have discrete functions, APS-1 sera have been shown to bind and neutralize all tested IFN-α paralogs. In this paper, we will refer to the tested protein, IFN-α A, as simply IFN-α.

Autoimmune polyendocrine syndrome type I is caused by mutations in the gene autoimmune regulator (AIRE). AIRE is a transcriptional regulator, expressed predominantly in medullary thymic epithelial cells, responsible for the expression of a broad array of peripheral tissue antigens (11, 12). Peripheral tissue antigens are presented to developing T cells in the thymus, and autoreactive T cells with high affinity T cell receptors are deleted (13). Thus, *AIRE* serves as a key gene in T cell negative selection. APS-1 patients mount autoantibodies to a variety of different antigens (14, 15), but why all APS-1 patients mount autoantibodies against type I IFNs with nearly 100% specificity is an enigma. The *in vivo* consequences of ACAAs against type I IFNs in APS-1 are equally unclear, as APS-1 patients do not suffer increased frequencies of viral infections. However, a recent study identified an inverse correlation between type I IFN ACAAs and the prevalence of type I diabetes (T1D) (16–18).

Adding to our knowledge of ACAAs in immunodeficiency, we recently discovered high titer type I IFN ACAAs in *RAG1* and *RAG2* mutation-associated immunodeficiencies (10). Lower titer IFN-α ACAAs have also been described in systemic lupus erythematosus and at lower prevalence in healthy individuals (3–5). The prevalence, etiology, and function of ACAAs in many other primary immunodeficiencies remain to be defined.

Immunodysregulation polyendocrinopathy enteropathy X-linked (IPEX) is a severe X-linked primary immunodeficiency and autoimmune disease caused by loss of function mutations in the gene *FOXP3* (19). Most patients present shortly after birth with fulminant autoimmunity, including most prominently autoimmune enteropathy, T1D, and eczematous dermatitis. *FOXP3* is a master regulator of T regulatory cells (T_reg_). In IPEX patients, T effector cells (T_eff_) can become activated in the absence of the tolerizing functions of T_reg_ leading to autoimmunity (20). IPEX patients are treated with pharmacologic immunosuppression, and bone marrow transplant has shown benefit for a subset of patients (21). Gene therapy trials aimed at editing the defective *FOXP3* locus are under development.

IPEX-specific autoantibodies against the peripheral autoantigens, villin, and harmonin have been described (22). To address whether ACAAs are present in IPEX, we performed a proteomic screen for ACAAs using custom-designed protein microarrays in a small cohort of IPEX patients. We discovered IgG ACAAs against IFN-α in IPEX sera and confirmed their presence by immunoassay. We extended our studies to a larger cohort of patients with IPEX and IPEX-like disease and also found elevated levels of IFN-α ACAAs in *post hoc* analysis. To understand the function of these ACAAs, we performed *in vitro* blocking experiments which demonstrate serum blocking activity correlating strongly with IFN-α ACAA titer. Last, we purified IgG from sera to formally demonstrate that serum blocking activity is contained in the IgG fraction. Taken together, these studies describe the first identification of ACAAs against type I IFNs in IPEX.

## Materials and Methods

### Patients

Samples from the European cohort were collected with informed consent under an ethical IRB approved at the San Rafaelle Hospital in Milan, Italy. Samples from the Seattle cohort were either collected with informed consent or de-identified under ethical IRB-approved protocols at the Seattle Children’s Hospital, where they were deemed exempt from requiring informed consent. For patients who went on to receive stem cell transplant, samples were collected before transplant. Post-transplant samples were processed when available, but excluded from the analysis. All APS-1 patients showed homozygous or compound heterozygous known or predicted pathologic mutations in *AIRE*. For all IPEX patients, sequencing of *FOXP3* revealed the presence of a known loss of function mutation. IPEX-like patients were defined as infants or children with the onset of autoimmune enteropathy or T1D mellitus in addition to another autoimmune feature with normal *FOXP3* sequencing. We excluded from our IPEX-like control group patients with an identifiable genetic defect, including mutations in CD25, STAT1, STAT3, LRBA, or CTLA-4. Polyautoimmunity patients included as controls were defined as infants or children with the onset in infancy of either (A) 2 autoimmune diseases plus autoimmune serology or family history, or (B) 3 autoimmune diseases. The European cohort consisted of 6 IPEX patients, 10 IPEX-like patients, 15 healthy controls, 5 APS-1 patients, and 24 polyautoimmunity patients. The Seattle cohort consisted of 19 IPEX patients, 12 IPEX-like patients, 6 APS-1 patients, and 9 healthy controls.

### Antigen Microarrays

Antigen microarrays were printed using a Bio-Rad ChipWriter Compact robotic microarrayer and ChipWriter Pro software. 24 biomolecules were purchased from multiple vendors and printed in triplicate at dilutions of 200 µg/ml onto epoxysilane-coated glass slides (Schott #1064016). A complete list of molecules and their vendors can be found in Table S3 in Supplementary Material.

Arrays were first blocked in 7% fetal bovine serum (FBS) (Omega Scientific) in PBS plus 0.1% Tween (Sigma-Aldrich) (PBST) for 1 h at 4°C rocking, and then washed three times in PBST. Arrays were probed for 1 h with serum diluted 1:150 in 30% FBS 1% PBST, rocking at 4°C. Arrays were subjected to three 5 min washes in 1% PBST. Serum reactivity was detected using an Alexafluor 647-conjugated goat anti-human IgG secondary antibody (Jackson #109-605-098) diluted to 2.5 µg/ml in 30%FCS 1% PBST for 45 min. Arrays were washed three times in PBST and dried under negative pressure.

Arrays were scanned using an Agilent microarray scanner and images processed using GenePix 6 software. Mean fluorescence intensities (MFIs) for each antigen were calculated by taking the mean of median fluorescence intensity for each replicate feature of each antigen.

### IFN-α ACAA Immunoassay

An indirect immunoassay was performed by coating Nunc MaxiSorp^®^ plates (catalog #44-2404-21) with IFN-α A (PBL, catalog #11101) at 1 µg/ml in PBS, blocking with 30% FBS in PBST, probing with serum diluted 1:100 in 5% FBS in PBST, and detecting with anti-human reagents purchased from Delfia per protocol (catalog #1244-330, www.perkinelmer.com).

### IgG Purification

IgG purification was performed using Pierce Protein G Spin kit per protocol (Thermoscientific #89949).

### IFN-α Blocking Assay

Frozen peripheral blood mononuclear cells from a healthy donor were thawed and 5 × 10^5^ live cells were aliquoted into polysterene tubes (BD #352052) in 100 µl of RPMI 1640 medium with 10% FCS and 1% penicillin-streptomycin (Gibco #15070063) and incubated at 37°C for 2 h. 11 µl of either serum or medium were added to tubes, which were vortexed and incubated for 15 min. Cells were stimulated with IFN-α A (PBL #11102-2) in a 10 ul aliquot to tubes for a final concentration of 10^3^ Units/ml. Cells were incubated for 15 min, then promptly fixed for 10 min with parafaormaldehyde at final concentration of 1.5% (Electron Microscopy Sciences #15710) at room temperature. Cells were then permeabilized with cold methanol at 4°C for 20 min and then washed in cold PBS. Cells were stained with the following antibodies: BV421 mouse anti-human CD3 (BD #562426), PerCP-Cy5.5 mouse anti-human CD4 (BD #341654), and PE Mouse Anti-STAT1 (pY701) (BD #612564). Cells were washed and data acquired on an LSR II cytometer (BD). For each blocking experiment, percent of cells in the pSTAT1 gate was normalized to set the highest percentage as 100% by dividing all samples by the percentage of the highest sample.

### Statistical Analysis

Heat maps were generated and significance analysis of microarrays (SAM) performed using the free software Multiple Experiment Viewer (23, 24). SAM false discovery rates (FDR) were set to <0.001.

Indirect immunoassay results were tested for statistical significance using the Mann–Whitney test. Correlations between microarray and immunoassay data, and between microarray and blocking data, were performed using a quadratic non-linear regression and linear regression, respectively, using Prism (Graphpad) software. Statistical significance of differences in blocking activity was calculated using an unpaired *t*-test.

## Results

### Antigen Microarrays Identify ACAAs

Anti-cytokine autoantibodies are prominent in APS-1, which is caused by mutations in *AIRE*, a master regulator of T cell central tolerance. We hypothesized that ACAAs would also be a feature of IPEX, caused by mutations in *FOXP3*, a master regulator of T_reg_, as IPEX patients have defects in peripheral T cell tolerance. We studied 6 patients with IPEX, 10 patients with IPEX-like disease, 24 patients with polyautoimmunity, and 5 patients with APS-1. The clinical characteristics of IPEX patients in this cohort included diverse manifestations of autoimmunity and immunodeficiency (Table S1 in Supplementary Material).

To discover novel ACAAs in primary immunodeficiencies and autoimmune diseases, we designed and fabricated protein microarrays with features, including cytokines, chemokines, growth factors, anti-microbial proteins, traditional autoantigens, and other biomolecules. After screening for autoantibodies in cohorts of other primary immunodeficiency patients, we selected 24 of the most promising candidate antigens and printed targeted protein microarrays for the study of IPEX patients. Microarrays were probed with diluted serum, scanned, and analyzed (Table S4 in Supplementary Material).

As expected, high reactivity autoantibodies against the type I IFNs; IFN-ω and IFN-α A (hereafter referred to as IFN-α); were detected in all APS-1 patients (Figure S1 in Supplementary Material). Serum derived from most, but not all APS-1 patients contained ACAAs against IL-17A and IL-17F, consistent with previously reported frequencies (25, 26). High and mid-range reactivity against the type III IFNs IL-28A and IL-28B were also observed in two patients. Our group and others have observed type III IFN ACAAs in small subsets of patients and in healthy individuals (unpublished observations J.M.R.). Autoantibodies against the traditional autoantigen thyroid peroxidase were also observed throughout the cohort and were most frequently found in patients with polyautoimmunity.

**Figure 1:**
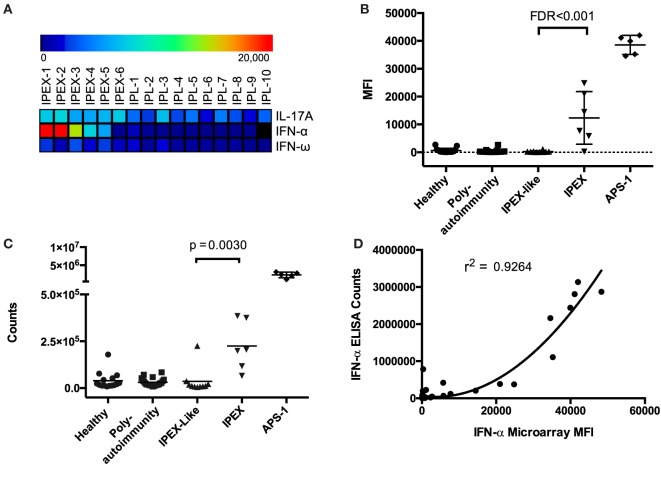
Antigen microarrays identify anti-cytokine autoantibodies (ACAAs). **(A)** Significance analysis of microarrays identifies ACAAs enriched in immunodysregulation polyendocrinopathy enteropathy X-linked (IPEX) compared to IPEX-like disease (IPL) (false discovery rates <0.001). **(B)** ACAA mean fluorescence intensity values against interferon-α (IFN-α) in all patients studied as measured by antigen microarray. **(C)** ACAA counts against IFN-α in all patients studied as measured by indirect immunoassay (*p* = 0.003 Mann–Whitney) **(D)** Correlation of IFN-α ACAA values as measured by microarray compared to indirect immunoassay (*r*^2^ = 0.924 using quadratic regression).

To identify autoantibodies unique to IPEX, we compared autoantibody levels between samples from IPEX and IPEX-like patients. After performing two-class SAM, we found statistically significant elevated levels of autoantibodies against IFN-α, IFN-ω, and IL-17A in IPEX samples as compared to IPEX-like samples (FDR < 0.001) (Figure 1A). ACAAs against IFN-α were of medium intensity compared to ACAAs in sera from APS-1 patients, while those against IFN-ω and IL-17A were low, all below 10,000 units MFI. To confirm differences between healthy controls as well, we also compared autoantibody levels between samples from IPEX and healthy controls and found the same three ACAAs statistically elevated (FDR < 0.001), in addition to seven other autoantibodies. Since lower intensity ACAAs are less likely to have clinical significance, and given the known association of IFN-α ACAAs in APS-1, we focused further experiments on the role of IFN-α ACAAs in IPEX. We did not test for reactivity against the 12 other IFN-α paralogs, but in APS-1 high levels of cross-reactivity to the paralogs have been demonstrated (9).

Interferon-α and IFN-ω ACAAs were found across a range of MFIs in IPEX samples, but all were at levels lower than those found in APS-1 (Figure 1B). We validated IFN-α ACAA microarray results by indirect immunoassay (*p* = 0.030) (Figure 1C). We correlated results between the two platforms and found highly significant correlation with *r*^2^ = 0.926 (Figure 1D).

### Blocking Activity of IFN-α ACAAs

**Figure 2:**
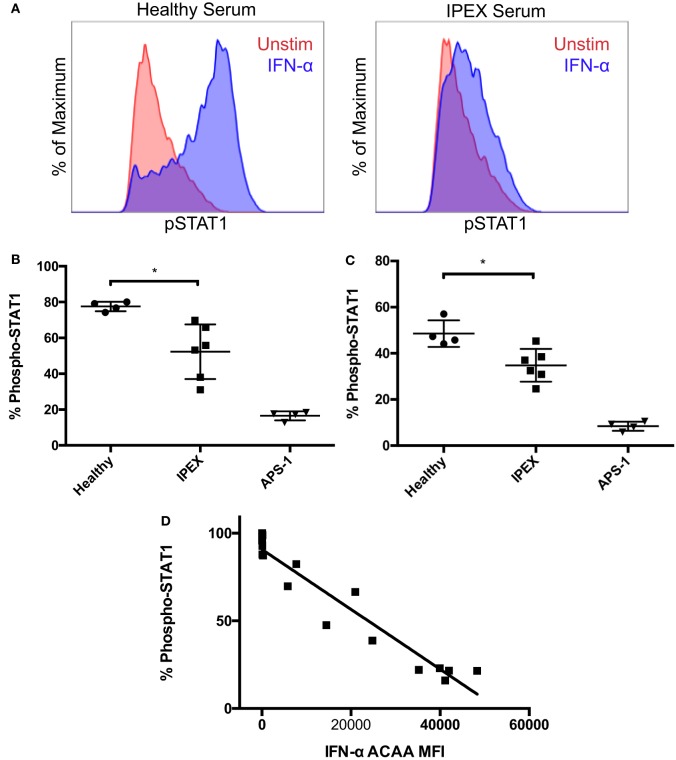
Blocking activity of interferon-α (IFN-α) ACAAs. **(A)** Aliquots of a healthy donor’s PBMCs were incubated with serum and either stimulated with IFN-α or left unstimulated. Representative flow cytometry plots measuring STAT1 phosphorlyation in CD4+ gated T cells are shown. **(B)** Percent of CD4+ T cells gated for STAT1 phosphorylation after IFN-α stimulation in the presence of healthy, IPEX, or autoimmune polyendocrine syndrome type I sera (*p* = 0.012) or **(C)** IgG purified from sera (*p* = 0.013). Unpaired *t*-test. **(D)** Correlation between IFN-α ACAA reactivity and blocking activity (*p* < 0.001, *r*^2^ = 0.925), linear regression.

To further investigate the functional activity of IFN-α ACAAs in IPEX, we tested sera from IPEX and control sera for IFN-α blocking activity *in vitro*. We tested serum for blocking activity by measuring its ability to inhibit IFN-α-induced STAT1 phosphorylation in heterologous CD4+ T cells (Figures 2A,B). As expected, sera from healthy donors had minimal blocking activity. Sera from APS-1 patients, which had the highest levels of IFN-α ACAAs, had very high blocking activity. Sera derived from IPEX patients, which had intermediate levels of IFN-α ACAAs, demonstrated intermediate levels of blocking.

To demonstrate that blocking activity was found in the immunoglobulin fraction, we purified IgG from serum samples using protein G beads. Purified IgG reproduced the same patterns of blocking activity compared to the sera from which they were derived (Figure 2C). Flow-through fractions of sera depleted of IgG lost their blocking activity (data not shown). These results demonstrate that the serum factor blocking IFN-α signaling *in vitro* is IgG immunoglobulin.

To understand the relationships between IFN-α ACAA titer and blocking activity, we correlated IFN-α ACAA MFI with *in vitro* blocking activity from the corresponding sample (Figure 2D). IFN-α ACAA MFI and blocking activity were highly statistically significantly correlated (*r*^2^ = 0.9246, *p* < 0.0001). Taken together, these results demonstrate that IFN-α ACAAs in IPEX are of the IgG class and block IFN-α in proportion to their binding activity.

### IFN-α ACAA Levels in a Second IPEX Cohort

To expand our observations to a second cohort, we measured IFN-α ACAAs in IPEX patients and IPEX-like patients without known causative mutations who were cared for at the Seattle Children’s Hospital. In the Seattle cohort, patients also had mutations in all four domains of *FOXP3* (Table S2 in Supplementary Material). Samples and control sera were tested by indirect immunoassay for IFN-α reactivity as described in Section “Materials and Methods.” Only pre-transplant samples were included in our analysis.

When comparing all IPEX patients to IPEX-like patients, IPEX patients had higher levels of IFN-α ACAAs, but this difference did not reach statistical significance, *p* = 0.078 (Figure S2A in Supplementary Material). On further examination of this cohort, we noticed significant age differences between IPEX-like and IPEX groups (7.91 v. 0.71 median years of age), which may represent a source of confounding. To control for this difference, we performed a *post hoc* analysis, including only patients greater than 1 year of age. In this *post hoc* analysis, we found elevated levels of IFN-α ACAAs in IPEX and this difference reached statistical significance (*p* = 0.007) (Figure S2B in Supplementary Material). We similarly compared IPEX patients to healthy controls. In the all ages group, there was no statistical difference between the groups (*p* = 0.530), and in the *post hoc* >1-year-old analysis, difference approached, but did not reach statistical significance (*p* = 0.0745). Surprisingly, two healthy control samples had medium level IFN-α ACAAs which contributed to a decrease in statistical significance, and further clinical history on these two individuals could not be obtained.

Separately, in this experiment we tested sera from IPEX-like patients with known causative mutation. One patient with a heterozygous gain of function *CTLA-4* mutations, a recently discovered cause of IPEX-like disease (27) had elevated IFN-α ACAAs when compared to IPEX-like patients without a known causative mutation. Similarly, five patients with *STAT1* gain of function mutations, another cause of IPEX-like disease, also had elevated levels of IFN-α ACAAs (*p* = 0.0365). To our knowledge, these are the first reports of IFN-α ACAAs in patients with *STAT1* and *CTLA-4* mutations. Given the small number of these patients tested, these observations should be tested for reproducibility in larger cohorts.

### Correlates of IFN-α ACAAs

In the two cohorts, patients had mutations in each of FOXP3’s four domains: the N-terminal, zinc-finger, leucine zipper, and forkhead domains. Although the number of samples was small, we did not see correlation between mutations in each domain and IFN-α ACAA titer. In fact, in the European cohort the sample with the highest reactivity came from a patient with a mutation in the N-terminal domain, and the second highest sample came from a patient with a mutation in the C-terminal domain (Table S1 in Supplementary Material).

The clinical consequences of IFN-α ACAAs *in vivo* are poorly understood (10). Type I IFNs are critical for antiviral immunity, but APS-1 patients with IFN-α ACAA are not known to suffer from more frequent viral infections. To assess for a phenotype of IFN-α ACAAs *in vivo*, we studied correlations between clinical and molecular phenotypes. The severity and frequency of viral infections, such as cytomegalovirus, herpes simplex virus, and *S. aureus* infection were more frequent than in the general population, but severity of opportunistic infection was not correlated with IFN-α ACAA titer (Tables S1 and S2 in Supplementary Material). There were no observed clinical infections after immunization with routine live vaccines. We also correlated age with IFN-α ACAAs in each cohort, and these correlations were not statistically significant.

A recent study has identified an inverse correlation between type I IFN ACAAs and T1D in APS-1 (18). In our IPEX cohort, we did not observe the same correlation as T1D was present in patients with both high and low reactivity IFN-α ACAAs (Table S1 in Supplementary Material). However, our study may not be powered to detect such differences, and the levels of reactivity in IPEX are significantly lower than those seen in APS-1, so effects seen by Meyer et al. may not be seen at lower titers.

## Discussion

These data taken together demonstrate that low titer IgG IFN-α ACAAs with blocking activity are a feature of IPEX. In a European cohort, we found elevated levels of IFN-α ACAAs in IPEX compared to both IPEX-like and healthy controls. In a second cohort from Seattle Children’s Hospital, we found elevated levels of IFN-α ACAAs in IPEX compared to IPEX-like patients in a *post hoc* analysis looking at children greater than 1-year-old. However, we do note that in the Seattle cohort differences between IPEX and all IPEX-like, and IPEX and healthy controls did not reach statistical significance. The lack of statistically significant difference between IPEX and healthy controls in this cohort may be due to unexpectedly elevated levels of IFN-α ACAAs in the healthy control group, which may represent random variation in the healthy population or undiagnosed disease. Given these findings, it will be informative going forward to test for reproducibility in other prospective cohorts of IPEX patients with both sufficient IPEX-like and healthy controls.

High titer IFN-α ACAAs are found in all patients with APS-1, which is caused by mutations in *AIRE*. Recent work has shown that high-titer type I IFN ACAAs are also found in most patients with RAG-mutation-associated primary immunodeficiency and in some cases of thymoma (10, 28). In both thymoma and immunodeficiency caused by *RAG1* or *RAG2* mutations, thymic deficiency of *AIRE* has been documented. This supports a unifying model, whereby defects in thymic *AIRE* expression are sufficient to generate type I IFN ACAAs at high titer and possibly complete penetrance (29). In contrast, less frequent and lower titer type I IFN ACAAs have been described in systemic lupus erythematosus, a complex disease characterized by defects in both central and peripheral tolerance (3–5, 30). Similarly, in IPEX, a disease with defects of peripheral T cell tolerance, we now report ACAAs at lower titers than found in APS-1.

AIRE is the best described regulator of the central T cell tolerance checkpoint. Similarly, *FOXP3* is required for peripheral T cell tolerance as a master regulator of T_reg_ (31). T_reg_ can also develop and reside in the thymus, so although IPEX’s major defect is that of peripheral tolerance, there may be a minor defect in central tolerance as well. These genes are mutated in APS-1 and IPEX, respectively. These genetic diseases, as experiments of nature, allow us to localize defects in immune tolerance. Using the development of IFN-α ACAAs as one measure of autoimmunity, our results suggest a model with a necessary role for central T cell tolerance and an important, but non-essential role for peripheral T cell tolerance. Our human studies are particularly revealing as type I IFN ACAAs are not observed in the murine model of APS-1 (32).

Two vexing questions remain. First, why are autoantibodies generated against such a specific target? *AIRE* activates the transcription of a panoply of peripheral tissue antigens [although in the mouse IFN-α is not among them (33)], so the reason why type I IFNs are so exquisitely targeted is not obvious. One possibility is that IFN-α is specifically targeted because of its high level of inducibility and expression in inflamed tissue. IFN-α is expressed by the large majority of cell types and tissues (ProteinAtlas.org and BioGPS.org), and in the thymus, 35% of dendritic cells are plasmacytoid dendritic cells, major producers of type I IFNs. Patients with IPEX have a failure of tolerance which leads to inflammation and enteropathy, and indeed this inflammation may cause higher levels of IFN-α. In normal states of immune tolerance, T cells with high affinity interactions to IFN-α epitopes would be expected to be deleted by negative selection. However, in organisms with defects of tolerance, high local IFN-α concentrations could trigger an autoimmunization event (17). Such an immunization event is most likely to occur in the context of a viral infection, during which plasmacytoid dendritic cells secrete large amounts of type I IFN locally and systemically. In support of a viral trigger, we previously found an association between past Varicella infection and the development of IFN-α ACAAs in patients with *RAG* deficiency (10). Our studies support such a model where defects in either central or peripheral tolerance can lead to failure to prevent IFN-α ACAAs, and tolerance to type I IFNs is maintained in a delicate balance by multiple checkpoints, each vulnerable to breach.

The second major remaining question is, what, if any, is the *in vivo* function or phenotype of these ACAAs, and are they helpful, harmful, or irrelevant to the host. In mice, genetic deficiency or passive transfer of blocking antibodies against type I IFNs increase susceptibility to viral infection (7, 34). However, APS-1 patients, although susceptible to Candida infection, have not been found to be more susceptible to viral infections (16). Similarly, in SLE patients, no major clinical or serological differences were observed between patients with or without IFN-α ACAAs (4). In our study, after analyzing clinical, cellular, and molecular data, we did not observe a correlation between IFN-α ACAAs and increased rates of viral infection.

Recent results of multiple phase II randomized placebo controlled trials utilizing IFN-α blockade for the treatment of SLE are germane to the question of the *in vivo* effect of IFN-α ACAAs (35, 36) (Astrazeneca ACR 2015). In all three trials, serious adverse events were similar between treated and placebo groups. However, all three trials showed increased rates of *Varicella zoster* reactivation in patients with IFN-α blockade. These results provide evidence that IFN-α ACAAs may increase the risk of viral infection, particularly *Varicella zoster* reactivation.

In summary, we have found elevated levels of IFN-α IgG blocking ACAAs in IPEX patients. These studies add to a literature of diseases in which IFN-α ACAAs have been described. The discovery of type I IFN ACAAs in IPEX, an experiment of nature, aids in the localization of defective tolerance checkpoints involved in the generation of type I IFN ACAAs. Their prevalence across immunological diseases demonstrates a primary importance for central T cell tolerance and a secondary role for peripheral T cell tolerance in preventing these ACAAs. Furthermore, given their prevalence in APS-1, *RAG1/RAG2*-associated immunodeficiencies, SLE, and now IPEX, it will be important to consider type I IFN ACAAs as potential diagnostics and biomarkers both in immunodeficiency and autoimmunity.

## Ethics Statement

Samples from the European cohort were collected with informed consent under an ethical IRB approved at the San Rafaelle Hospital in Milan, Italy. Samples from the Seattle cohort were either collected with informed consent or de-identified under ethical IRB-approved protocols at the Seattle Children’s Hospital, where they were deemed exempt from requiring informed consent.

## Author Contributions

JR designed and performed the experiments and wrote the manuscript. PU and RB designed experiments and assisted in the writing of the manuscript. MM, FB, EA, CP, GW, TT, and RB supplied valuable clinical samples.

## Conflict of Interest Statement

The authors declare that the research was conducted in the absence of any commercial or financial relationships that could be construed as a potential conflict of interest. The reviewer ML and handling editor declared their shared affiliation.

## Supplementary Material

Figure S1 Total antigen microarray data. Anti-cytokine autoantibodies mean fluorescence intensity from total array data are represented in heat map format with samples along the *x*-axis and antigens along the *y*-axis.

Figure S2 **(A)** Interferon-α ACAA levels in a second immunodysregulation polyendocrinopathy enteropathy X-linked cohort from Seattle, Washington as measured by indirect immunoassay including all samples and **(B)** *post hoc* analysis including only samples >1-year-old.

Table S1 Immunodysregulation polyendocrinopathy enteropathy X-linked European Cohort Clinical and Laboratory Characteristics. Abbreviations: IS, immunosuppression; T1D, type I diabetes; HSCT, hematopoietic stem cell transplantation; ITP, immune thrombocytopenic purpura; CMV, cytomegalovirus; HSV, herpes simplex virus.

Table S2 Immunodysregulation polyendocrinopathy enteropathy X-linked Seattle Cohort Clinical and Laboratory Characteristics.

Table S3 Antigen vendors.

Table S4 Protein microarray raw data.
