# Supplementary figures and images for: Neutralizing Anti-Cytokine Autoantibodies Against Interferon-α in Immunodysregulation Polyendocrinopathy Enteropathy X-Linked
Source: Front Immunol. 2018 Mar 29;9:544. doi: 10.3389/fimmu.2018.00544 (PMC5885158; doi:10.3389/fimmu.2018.00544)

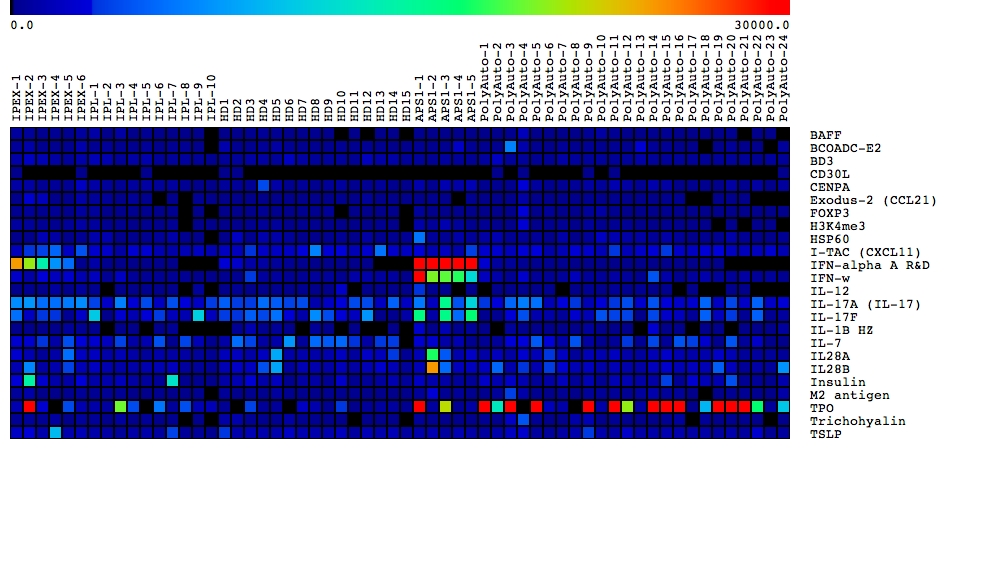

Supplement: Figure S1 — Total antigen microarray data. Anti-cytokine autoantibodies mean fluorescence intensity from total array data are represented in heat map format with samples along the x-axis and antigens along the y-axis. [file Image_1.jpeg]

a.

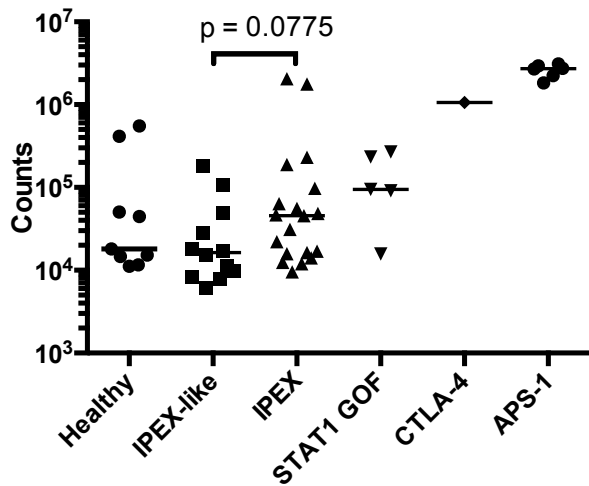

b.

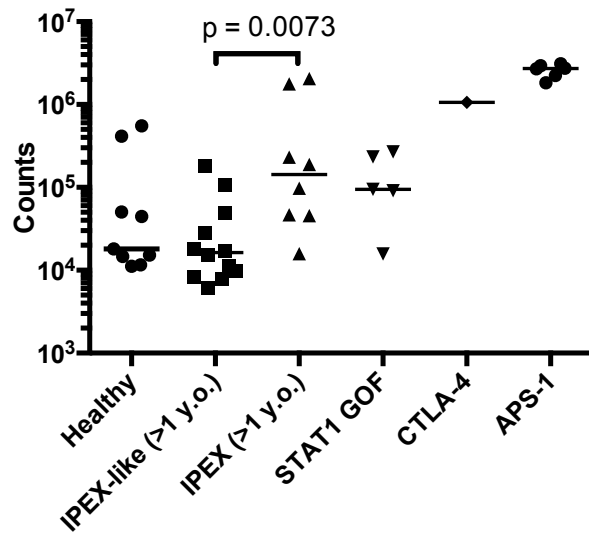

Supplement: Figure S2 — (A) Interferon-α ACAA levels in a second immunodysregulation polyendocrinopathy enteropathy X-linked cohort from Seattle, Washington as measured by indirect immunoassay including all samples and (B) post hoc analysis including only samples >1-year-old. [file Image_2.PDF]
