# Supplementary material for: Neutralizing Anti-Cytokine Autoantibodies Against Interferon-α in Immunodysregulation Polyendocrinopathy Enteropathy X-Linked
Source: Front Immunol. 2018 Mar 29;9:544. doi: 10.3389/fimmu.2018.00544 (PMC5885158; doi:10.3389/fimmu.2018.00544)
Supplement: Table S1 — Immunodysregulation polyendocrinopathy enteropathy X-linked European Cohort Clinical and Laboratory Characteristics. Abbreviations: IS, immunosuppression; T1D, type I diabetes; HSCT, hematopoietic stem cell transplantation; ITP, immune thrombocytopenic purpura; CMV, cytomegalovirus; HSV, herpes simplex virus. [file Table_1.PDF]

IPEX Sample #	Age (years)	FoxP3 Mutation	IFN-α MFI by Microarray	IFN-α Counts by Immuno-assay	Infectious Complications	Autoimmune Manifestations	Therapy
1	10	c.210+1 G>A	24809	374904	No opportunistic infection.	Enteropathy	IS
2	15	c.1150G>A	21000	384644	CMV and enteritis at 8y. P. jirovecii pneumonia at 11y while under immunosupression.	Enteropathy, severe eczema, T1D, thyroiditis, alopecia, autoimmune hemolytic anemia	IS
3	28	c.816+2del	14486	205781	Severe bronchial infection and osteomyelitis in first year of life. S. aureus sepsis and HSV at 17y while on immunosuppression.	Enteropathy, Eczema, ITP, polyarthritis	IS
4	3.4	c.750-752del	7725	117273	No opportunistic infection.	Enteropathy, Eczema, ITP	IS, HSCT
5	11	c.1040 G>A	5831	65946	No opportunistic infection.	Enteropathy	IS
6	10	c.1040 G>A	243	199066	No opportunistic infection.	Enteropathy, T1D, eczema, ITP, hepatitis	IS
